# Assessing atmospheric nitrogen losses with photoacoustic infrared spectroscopy: Polymer coated urea

**DOI:** 10.1371/journal.pone.0204090

**Published:** 2018-09-18

**Authors:** Joshua J. LeMonte, Von D. Jolley, Tobin M. Story, Bryan G. Hopkins

**Affiliations:** Department of Plant and Wildlife Sciences, Brigham Young University, Provo, UT, United States of America; Institute of Soil Science, CHINA

## Abstract

Although N is beneficial and essential for life, it is also a common atmospheric pollutant as nitrous oxide (N_2_O) and ammonia (NH_3_)—contributed largely from N fertilization. Polymer-coated urea (PCU) fertilizer is a promising controlled release fertilizer that provides improved N-release timing. Glasshouse studies were conducted to compare N_2_O and NH_3_ emissions from PCU and uncoated urea to an untreated control utilizing a non-static, non-flow-through chamber in conjunction with photoacoustic infrared spectroscopy (PAIRS) for gas collection and analysis. Three short-term 20-Day Studies with sand, sandy loam, and loam soils and a full-term 45-Day Study with loam soil were completed. Volatilization of NH_3_ was reduced by 72% and 22% in the sandy loam and loam soils, respectively, in two of the short-term studies and by 14% in the loam in the full-term study. Evolution of N_2_O was reduced by 42% and 63% in the sandy loam and loam soils of the short-term studies and by 99% in the loam soil of the full-term study. No differences were observed in the sand soil. Overall, PCU decreased gaseous losses of N following fertilization while providing a steady supply of N to the plant. Higher temporal resolution was observed with the PAIRS instrumentation as compared to what is typically reported and, as such, we recommend PAIRS analysis as a viable method for studying N gas emissions.

## Introduction

Despite N being beneficial and critical to life, its anthropogenic use has made it a common pollutant in the atmosphere and hydrosphere. Nitrogen may be lost as it is evolved as ammonia (NH_3_) or nitrous oxide (N_2_O) gases during natural conversion processes or lost as nitrate (NO_3_^-^) through surface runoff/erosion or from the rooting zone through leaching to groundwater. Nitrate in drinking water is a potential toxin to humans and other mammals if concentrations become excessive and high N in surface waters can lead to eutrophication and resultant death of a variety of aquatic species [1].

In terms of atmospheric pollution, Schlesinger and Hartley (1992) estimated that 10% of all manufactured N fertilizer worldwide is volatilized as NH_3_ gas[2]. Volatilization of surface-applied N fertilizers reached an excess of 60% over the first 10 days following fertilization in a growth chamber study using warm-season bentgrass (*Agrostis palustis* cv. Huds)[3]. Volatilized NH_3_ gas from fertilizer application is an environmental concern. Ammonia is more likely to deposit on land or water bodies through either wet or dry deposition than other forms of anthropogenic N. Such deposition in sensitive ecosystems can lead to soil acidification and surface water eutrophication [4,5]. Deposition in sensitive ecosystems can also lead to plant community loss and reduction of biodiversity [4]. Fenn et al. reported that increased NH_3_ deposition in typically N-limited ecosystems across the globe is having unwanted consequences, including increased aluminum mobility and resulting forest decline [6]. Increased levels of atmospheric NH_3_ also negatively affect air quality by contributing to smog formation [7].

Elevated emissions of N_2_O are even more concerning than NH_3_ volatilization. On average, it is conservatively estimated that 1% of all N applied as fertilizer in both organic and inorganic forms is lost to the atmosphere as N_2_O [8]. The actual amount emitted is directly related to the type, quantity, and method of application of fertilizer [8]. Hirsch et al. (2006) estimated that anthropogenic emissions of N_2_O have increased by approximately 50% over pre-industrial levels [9]. It is also estimated that 78% of the total annual anthropogenic N_2_O losses are related to fertilization, with automobile and industrial pollution contributing most of the remainder [10]. The concerns are that N_2_O is a long-lived, potent greenhouse gas with a global warming potential 310 times greater than carbon dioxide (CO_2_) and that it catalytically destroys ozone (O_3_) in the troposphere [10,11]. Emissions of N_2_O to the atmosphere via denitrification and nitrification are controlled by interacting factors such as soil aeration, temperature, texture, ammonium (NH_4_^+^) and NO_3_^-^ concentrations, and the microbial community [12].

Minimizing N losses and maximizing plant uptake conserves non-renewable resources and minimizes environmental impacts. Effectiveness of N uptake by plants is expressed as N use efficiency (NUE) [1]. Optimizing N fertilizer rate, source, timing, and placement are all essential to reach an ideal balance between economic profitability and NUE. One method of increasing NUE from a fertilizer source approach is to use controlled-release N (CRN) or slow-release N (SRN) fertilizers that release N into the soil over an extended period of time, ideally matching plant need and possibly reducing or eliminating labor-intensive and costly in-season N applications [1]. It is hypothesized that controlling N release will increase N retention and N uptake and thus mitigate N inefficiencies and losses to the environment [1,12,13].

Polymer-coated urea (PCU) fertilizer is a CRN that potentially provides improved N release timing. The rate of N release from PCU is temperature controlled, which protects N during periods of cool soil temperatures and slow plant growth and releases N as soil temperatures, plant growth, and N demands increase [1]. PCU’s can steadily supply the plant with N for longer periods of time following fertilizer application than immediately soluble forms of N, thus enhancing NUE and leading to increased crop yield and quality [1,3,13–19]. Hyatt et al. showed that the slower release of PCU can improve economics by eliminating additional in-season N applications [16]. Strong evidence supports PCU’s ability to mitigate negative environmental impacts associated with N fertilizer [13,20–22].

Polymer-coated urea has been shown to significantly decrease both NO_3_-N leaching and NH_3_ volatilization [3,7,13,20,22–27]. Use of PCU can also decrease nitrous oxide emissions [13,16,21,28,29].

Appropriate methods of analysis must be used to assess N_2_O and NH_3_ emissions from soils under unique N management regimes. Due to temporal and spatial variability and the multi-faceted nature of N_2_O emissions, reliable estimates are difficult to obtain. The widely used static chamber flux technique takes samples from small areas (< 1 m^2^) and is relatively inexpensive, although labor intensive if large numbers of samples are taken. However, sampling (typically done as three or more samples per chamber in 15- to 20-minute intervals) and analysis using a gas chromatograph (GC) with an electron capture detector increase temporal variability and decrease ability to estimate total losses over time. Venterea et al. estimated N_2_O emissions ranged from -60 to 170% of the mean [30]. Increasing the sampling frequency is needed to overcome such variability [31]. Photoacoustic infrared spectroscopy (PAIRS) is a recently developed method that allows in-field analysis of N_2_O concentrations. Advantages of PAIRS include analysis frequency, portability, robustness, and relative ease of use [32].

Another advantage of PAIRS is that it is easily configured to successively analyze NH_3_. Technological advancements have made capturing and analyzing NH_3_ more feasible and convenient. The improved denuder system allows monthly sampling to establish long-term trends [4]. Low-cost passive sampling is another method, which allows assessment of spatial variability of NH_3_ without active air sampling with samples taken daily [4].

Application of PAIRS in combination with non-static, non-flow-through chamber collection techniques provides a system of NH_3_ analysis that allows low maintenance, high frequency (multiple samples daily) automated sampling and monitoring [4]. Increasing the frequency of sampling should decrease temporal variation and provide increased understanding of NH_3_ emissions following fertilizer-N applications.

Those studies that have implemented PAIRS have commonly done so while utilizing either static chamber or flow-through systems [32]. While incorporating PAIRS analysis into these accepted collection methods has increased the number of sampling events and thereby decreased temporal variability, these collection methods can be expensive to automate and laborious to operate. A non-static, non-flow-through chamber method that adequately collects evolved gases while remaining open to the atmosphere would minimize some of the associated costs and sampling labor.

One objective of these studies is to develop such a novel and needed methodology for more accurately, conveniently, and completely measuring N pollutant gases. Another objective is to assess the differences in evolution of N_2_O and volatilization of NH_3_ from adjacent areas receiving N from two sources (urea and PCU) and rates using a simple non-static, non-flow-through chamber in conjunction with PAIRS analysis for continuous gas measurement and recording. The ultimate purpose in this work is to address the serious global problems caused by atmospheric contamination from anthropogenic N pollution.

## Materials and methods

### 20-Day studies

Three glasshouse studies were conducted at Brigham Young University (BYU, Provo, Utah, N 40° 14′41.35″ W 111° 38′ 57.42″, 1381 m). Other than soil texture (Table 1) and time of year, each study was nearly identical. The first study (Loam) was conducted from 14 Nov through 3 Dec 2008 (20 d) on a Timpanogos loam soil collected from the BYU research station (N 40° 4′1.77″ W 111° 37′ 44.99″, 1381 m). The second study (Sand) was conducted from 16 Dec 2008 through 9 January 2009 (20 d) on a manufactured sand soil (obtained from inert crushed quartz from a quarry in Emmett, ID, USA). The third study (Sandy Loam) was conducted from 24 Jan through 13 Feb 2009 (18 d) with a composited soil comprised of a 50/50 mixture of the manufactured sand and the Timpanogos loam. This study was ended after 18 d instead of the planned 20 d because of an instrument malfunction. The loam soil was air-dried, rocks removed, and soil clods larger than 4 cm diameter broken up and mixed thoroughly—minimizing pulverization of the soil’s structure (sand soil did not require drying or removal of rock).

**Table 1:** Soil properties for four N fertilization gas emission studies.

Studies	Soil Texture	Sand	Silt	Clay	Organic Matter	NO_3_^-^ -N	pH
		-—-—-—-—-—%—-—-—-—-—-				mg kg^-1^	
20 d	Sand	87	9	4	0.5	2	7.2
20 d	Sandy Loam	58	25	17	2.3	4.9	7.1
20 & 45 d	Loam	29	41	30	4	7.8	7.1

Treatments included: 1) 0 kg N ha^-1^ (control), 2) 336 kg N ha^-1^ urea (Urea-336; 46-0-0), 3) 224 kg N ha^-1^ urea (Urea-224) and 4) 224 kg N ha^-1^ PCU (PCU-224; 44-0-0; Environmentally Smart N, ESN, Agrium Advanced Technologies, Loveland, CO, USA). The physical properties of the fertilizers are: green and white color, 27 and 19.6 degrees angle of response, 0.80 and 0.77 Mg m^-3^ bulk density, 133 and 134°C melting point, 0.08 and 0.0000013 kPa [20°C] vapor pressure, 280–300 and 290–310 size guide number (SGN), and 67 and 63 uniformity index for urea and ESN, respectively. The particle size distribution for urea is 10.0, 59.0, 93.0, 99.0, and 100.0% for retention on sieves with 3.35, 2.80, 2.36, 2.00, and 1.70 mm mesh. The particle size distribution for ESN is very similar to the urea with 23.0, 80.0, 97.0, 99.7, and 100.0% over the screen sizes listed above. The chemical properties of the fertilizers are: 46 and 44% total N, 46 and 44% urea-N, 1.0 and 0.8 and 1.0% biuret concentration, 1.2 and 1.6% conditioner (methylene di-urea), 0.10 and 0.08% moisture, 8.0 and 7.2 pH of aqueous solution (10% wt./wt.), and 50 and 2 ppm free NH_3_ for urea and ESN, respectively. The aqueous release analysis for ESN was estimated at 40 d minimum and 90 d maximum for 80% N release at 23°C. Additionally, the coating weight for the ESN was 3.3% (all of the above percentages are on a weight basis). The coating is comprised of castor oil, polymer with polymethylenepolyphenylene isocyanate. The urea underlying the coating in ESN is identical to the urea that was used in this study—the only difference being the polymer coating.

Three replications of these four treatments were applied to soil in pots arranged in a randomized complete block design (RCBD). The 336 kg N ha^-1^ rate of urea was based on the estimated maximum agronomic rate of N for maize. The 224 kg N ha^-1^ rates of urea and PCU were based on preliminary field data suggesting a NUE of PCU to be approximately 33% better than uncoated urea [1]. Because of analytical instrument limitations (see below), a maximum of 12 experimental units (four rates and three replications) was used and PCU could not be evaluated at 336 kg N ha^-1^.

Twelve vented non-static, non-flow-through chambers were constructed out of standard 19 L polyvinyl chloride (PVC) buckets (30-cm diameter x 37-cm height). The bottom of each chamber was vented to allow water to move freely out and air to move freely in or out of the soil while simultaneously retaining the soil using a landscape fabric liner (Weed-barrier 1 oz., DeWitt Company, Sikeston, MO, USA). Chamber lids were constructed from fitted standard 19 L PVC bucket lids, sealed onto each chamber. A hole was cut in the center of each lid to accommodate a 13-cm diameter by 12-cm length PVC pipe sealed to the lid with acrylic latex silicone caulk. The PVC pipe reached to within 1–3 cm of the soil surface and to 2–3 cm above the lid. This was designed to minimize gas contamination from one chamber to another and to maximize accumulation of soil-evolved gases in the headspace in the chamber and yet allow gas exchange and equilibration with the atmosphere.

Each chamber contained approximately 13.5 kg of soil filled to within 8–12 cm of the top of the PVC container. Maize (Pioneer 35F38) was hydroponically germinated and four individual plants with shoots 8–12 cm high were transplanted into soil in the chambers with the plants extending up through the PVC pipe. On day one of each trial, all fertilizer treatments were applied to the soil surface, mixed into the top 5 cm of soil, and soils were immediately planted to maize, irrigated with municipal water (EC ≤280) to saturation, and gas sampling initiated. Targeted diurnal and nocturnal air temperatures were 25^o^ and 15°C, respectively, although limitations in heating/cooling capacity resulted in ranges of 20–29°C diurnally and 11–16°C nocturnally. Natural light was supplemented with artificial light (Hubbell high-pressure sodium lamps; Hubbell Inc., Greenville, SC, USA) to maintain a 14/10-hour light/dark cycle.

Soil volumetric water content was monitored using Watermark Soil Moisture Sensors (Spectrum Technologies, Plainfield, IL, USA) and logged using an AM400 soil moisture data logger (MK Hansen, Wenatchee, WA, USA), and soil temperature was monitored and logged using a common thermistor and the AM400 data logger. One pot per block was monitored. Due to the small plant size, soil volume, and chamber design minimizing evapotranspiration, the soil moisture remained between field capacity and maximum allowable depletion and at no point following the initial irrigation did any of the 20-Day Studies require irrigation. The soil moisture data shows that the conditions between pots of each treatment were essentially identical and, thus, not a factor in the study. If the soil had been saturated, we would have expected an increase in N_2_O emission rates, but the objective our studies was to conduct these trials under more typical field conditions.

Gas samples to measure emission of N_2_O and volatilization of NH_3_ were collected using an Innova 1309 12-port sampling unit (multiplexer) and analyzed with an Innova 1412 Photoacoustic Field Gas Analyzer (Lumasense Technologies, Santa Clara, CA, USA), via photoacoustic infrared spectroscopy (PAIRS) [32]. Gas samples were transported from the headspace above the soil using a pump on the PAIRS unit to pull the sample through 6 m of 4 mm ID HDPE tubing to the detector. The PAIRS unit was connected to a computer that controlled gas sampling (N_2_O and NH_3_) time intervals and analysis. A complete sample set of 12 pots was automatically sampled every 30 minutes 24 hr day^-1^—sampling continuously throughout each experiment.

### 45-Day study

Another study on the Timpanogos loam soil was conducted to evaluate NH_3_ and N_2_O losses over the full term of N release anticipated with PCU. Duration CR45 (44-0-0; Agrium Advanced Technologies, Loveland, CO, USA) is the PCU used in this study. This PCU, primarily used in the turfgrass and greenhouse markets, has a nearly linear release of N over 45 days, whereas ESN used in the 20-Day Studies is an agronomic product designed with an S-shaped release curve to match typical crop demand for N over 60–75 d (Alan Blaylock, Agrium Advanced Technologies, personal communication).

This study was also conducted in the BYU glasshouse from 13 Dec 2010 to 27 Jan 2011 (45 d). Treatments included: 1) 0 kg N ha^-1^ (control), 2) 200 kg N ha^-1^ urea (Urea-200), and 3) 200 kg N ha^-1^ PCU (PCU-200). Four replications of these three treatments were applied to soil with containers arranged in a RCBD. Fertilizer treatments, planting, and irrigation were managed the same as the 20-Day Studies.

Twelve chambers were constructed as described previously, with the following modifications. The PVC tube was pushed 5 cm into the soil and had four equally spaced holes, 5 mm in diameter, drilled through the tube wall 5–8 cm from the soil interface to maximize accumulation of soil evolved gases in the head space in the chamber, yet also allow gas exchange and equilibration with the atmosphere.

Soil volumetric water content and temperature were monitored identically to the 20-Day Studies. Unlike the 20-Day Studies, the soil did dry down in this study due to the longer time for evapotranspiration and, as such, all pots were irrigated via capillary upward flow to the point of field capacity based on the soil moisture readings.

Collection and analysis of N_2_O and NH_3_ emissions were completed in a nearly identical fashion as the earlier mentioned studies utilizing PAIRS analysis, although the sampling interval was shortened from 30 to 11 min for a higher temporal resolution.

### Gas data analysis

Concentrations, as determined by the PAIRS analyzer, were obtained, compiled, and used to determine daily flux for each chamber in each study. Daily flux was determined by using the maximum daily concentration and the minimum daily concentration that occurred prior in that day to the maximum concentration. Flux was then calculated by:

$$f=\frac{V\times \Delta C}{A\times \Delta t}$$

where *V* is the headspace volume, *ΔC* is the change in concentration (C_max_−C_min_) of N_2_O or NH_3_ as measured by PAIRS, *A* is the soil surface area of the chamber, and *Δt* is the time elapsed between C_min_ and C_max_. When determining *V* and *A*, the 13 cm diameter center used for plant growth that was open to the atmosphere was not included in the calculations for area and volume. The daily gas concentrations were used to determine the changes in time and concentration for input to the above equation. Concentrations were corrected using the ideal gas law, assuming standard temperature and pressure. Because this flux value was taken over an extended time (in some cases over six hours), it was assumed to be representative for that given day. To determine mass of N lost, daily flux values were assumed constant for a 24-h period.

Daily gas concentrations were statistically analyzed using ANOVA (SAS Institute, 2007). Loss of N as NH_3_ and N_2_O were then analyzed for statistical significance by ANOVA and Tukey HSD analyses with α = 0.05 (SAS Institute, 2007).

## Results and discussion

### Ammonia

#### 20-Day studies

**Fig 1:**
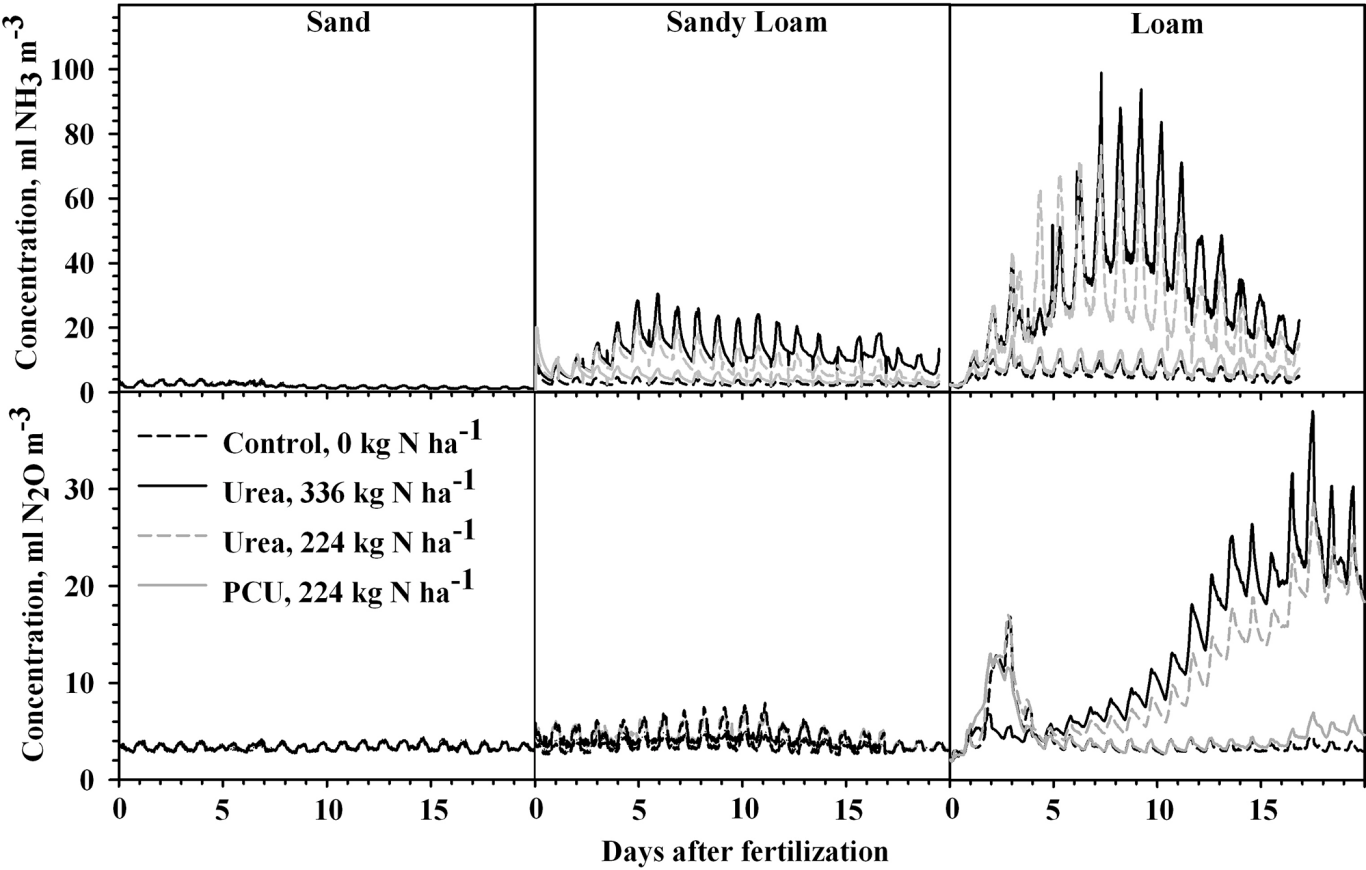
Concentration of NH_3_ and N_2_O in chamber headspace above soil fertilized with either uncoated urea (full and reduced rate) or polymer coated urea (PCU; reduced rate only) and compared to an untreated control in three 20 d studies with varying soil types. Statistical comparison of treatments was performed for each day (Table 2). Data for NH_3_ and N_2_O for d 18–20 of Sandy Loam study were lost due to instrument malfunction.

The fertilizer source by time interaction in the 20 day experiments was significant and therefore daily measurements of NH_3_ concentrations are presented for each treatment for the three soils (Fig 1).

**Fig 2:**
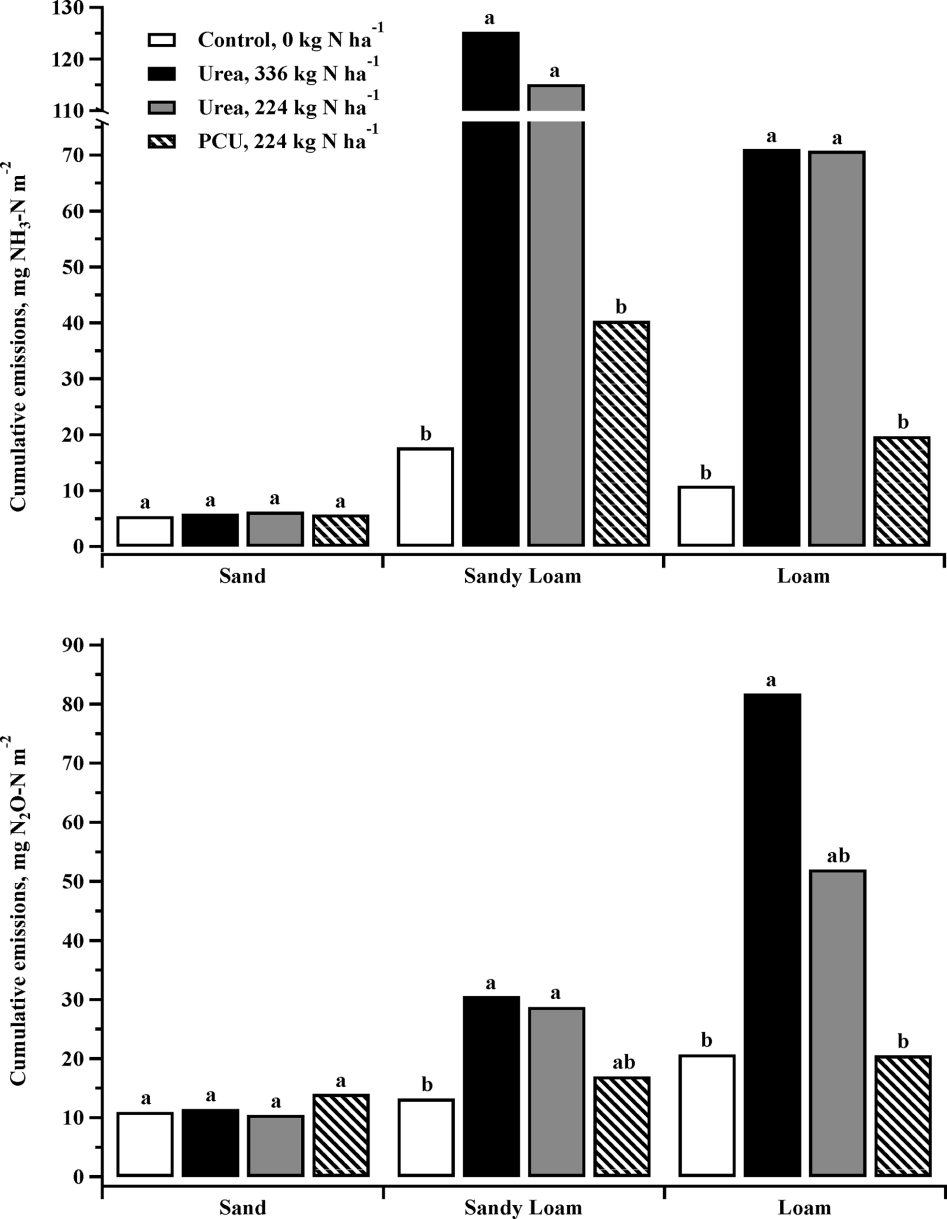
Estimated total emissions of NH_3_ and N_2_O from soil fertilized with either uncoated urea (full and reduced rate) or polymer coated urea (PCU; reduced rate only) and compared to an untreated control in three 20 d studies with varying soil types.

Sand Study–There were no significant differences in overall accumulated NH_3_ flux among the three treatments in the sand soil (*P* = 0.3304; Fig 2). The extremely low NH_3_ concentrations and fluxes observed are likely due to the sand soil being biologically inert and consequently having a low activity/level of the urease enzyme. Microbial enumeration analysis showed an unusually low microbial population (70,000 and 0 microorganisms g^-1^, respectively) and confirms the hypothesis that this sand soil was nearly biologically inert [33].

Sandy Loam Study–For the sandy loam soil, there were significantly greater NH_3_ concentrations evolved from both rates of urea than from PCU-224 and control treatments on all but days 1, 2, 5 and 6 (Fig 1 and Table 2). During the initial two days, concentration levels remained near background levels for all treatments. The NH_3_ concentration of the two rates of urea also differed significantly from each other often—generally Urea-336 produced significantly higher NH_3_ than Urea-224 (days 9–18). Only on two days (17–18) was NH_3_ evolution from PCU-224 significantly higher than the control. Thus, PCU was generally effective in minimizing NH_3_ volatilization in this sandy loam soil. Similar to another study done with maize in a sandy loam soil, elevated NH_3_ volatilization did not appear until 2 d after fertilization for both rates of urea (Fig 1) in the Sandy Loam study [34]. This is likely due to a relatively slow increase in soil pH in areas immediately adjacent to the fertilizer granules [35]. Following this increase in soil pH, conditions for NH_3_ volatilization were optimized and flux levels elevated. Volatilization from urea reached its peak at 6 d and gradually dropped toward background levels, although it remained higher than that of PCU-224 and control for the duration of the study (Fig 1).

**Table 2:** Statistical comparison of average daily concentration of NH_3_ and N_2_O in chamber headspace above soil fertilized with either uncoated urea (full and reduced rate) or polymer coated urea (PCU; reduced rate only) and compared to an untreated control in three 20 d studies with varying soil types and a 45 d study in loam soil.

Study	Soil Texture	————-—N Rate, kg ha^-1^ —————				Days Observed, daf^†^	Days Observed, %
		0	224 or 200^‡^ (CU)	224 or 200^‡^ (urea)	336 (urea)		
——————————————————NH_3_ ——————————————————							
20-Day Studies	Sand	A	A	A	A	1–20	100
	Sandy Loam^§^	C	C	B	A	9–16	44
		B	B	A	A	3–4, 7–8	22
		D	C	B	A	17–18	16
		A	A	A	A	1,6	11
		B	AB	A	A	2	5
		B	B	AB	A	5	5
	Loam^¶^	D	C	B	A	5–8, 14–20	69
		C	C	B	A	9–13	31
45-Day Study	Loam	B	B	A	n/a^#^	1, 5–18, 20–26	49
		A	A	A		31–45	33
		B	A	A		4, 27–30	11
		A	B	A		2	2
		A	B	AB		3	2
		B	C	A		19	2
——————————————————N_2_O ——————————————————							
20-Day Studies	Sand	A	A	A	A	20	100
	Sandy Loam^§^	B	B	A	A	3–4, 7–18	78
		C	B	A	A	1,6	11
		A	A	A	A	2	5
		D	C	B	A	5	5
	Loam^¶^	C	C	B	A	6–18	65
		D	C	B	A	19–20	15
		B	B	A	A	5	5
45-Day Study	Loam	C	B	A	n/a^#^	3–11	20
		B	B	A		12–45	76
		A	B	A		1	2
		B	C	A		2	2

† Days after fertilization

‡ 20-Day Studies fertilized with 224 kg N ha-1; 45-Day Study fertilized with 200 kg N ha-1.

§ Days 18–20 were not included for the 20-d sandy loam study due to instrument malfunction.

¶ Days 1–4 were not included for the 20-d loam study due to sample contamination from repairing chambers.

# The 45 d loam study did not include the high rate of urea.

Total cumulative N loss from NH_3_ volatilization (daily summation of NH_3_ measured from the first to the last day of the study) was also significantly higher for Urea-224 and Urea-336 than both PCU-224 (*P* = 0.0116 and 0.0229, respectively) and the control (*P* < 0.01, Fig 2). There was no difference between the two urea treatments (*P* = 0.9546) or between PCU-224 and the control (*P* = 0.678). Fertilization with 224 kg N ha^-1^ PCU rather than 224 or 336 kg N ha^-1^ urea reduced the average NH_3_ volatilization by 66% (Fig 2).

Loam Study–Significantly higher NH_3_ concentrations from volatilization were observed from the loam soil for both urea treatments than the PCU and control treatments (Fig 1 and Table 2). The only exceptions were days 1–4, days on which samples were contaminated from chemicals associated with a leak repair in some chambers. Ammonia concentrations from PCU-224 were significantly higher than the control on all other days excluding days 9–13.

Cumulative NH_3_-N volatilization loss from the control was significantly lower than both the Urea-336 and Urea-224 treatments (*P* = 0.0169 and 0.0173, respectively, Fig 2). There was no difference between the two urea treatments (*P* = 0.9867) or between PCU-224 and the control (*P* = 0.6697). Cumulative volatilization of both Urea-336 and Urea-224 were significantly greater than that of PCU-224 (*P* = 0.0334 and 0.0343, respectively). Fertilization with 224 kg N ha^-1^ PCU rather than 224 or 336 kg N ha^-1^ urea resulted in a 72 percent reduction in N lost via NH_3_ volatilization in this loam soil (Fig 2).

Appreciable volatilization occurred a day earlier from the loam than from the sandy loam soil. Volatilization peaked at 6 d for Urea-224 and at 7 d for Urea-336 treatment (Fig 1) and thereafter levels waned and trended toward background levels until the end of the study. Blaise and Prasad (1995) performed laboratory studies in which urea and PCU were surface applied to a bare sandy loam soil and found significant reductions (up to 53%) in NH_3_ loss by using PCU in both aerobic and anaerobic conditions [36]. Our findings in the Sandy Loam and Loam studies were in line with their findings. Overall, NH_3_ volatilization was reduced by 66% and 72% in the sandy loam and loam soils, respectively, with PCU compared to urea. These reduction rates are similar to those found by others [37,38]. This reduction may be attributed to the controlled N release by the polymer coating, so that the N supply matches the biological demand.

#### 45-Day study

**Fig 3:**
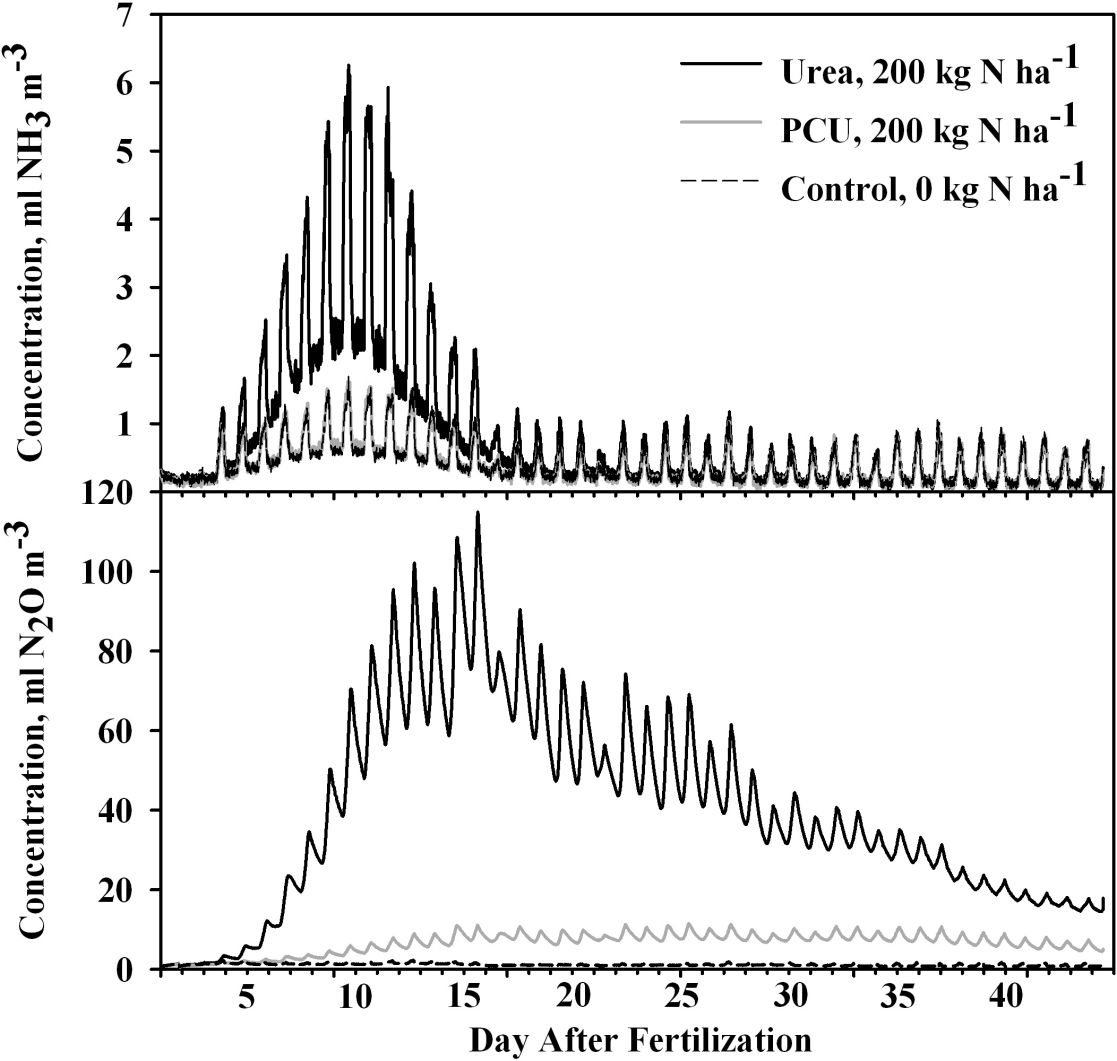
Concentration of NH_3_ and N_2_O in chamber headspace above soil fertilized with either uncoated urea or polymer coated urea (PCU) and compared to an untreated control in a 45 d study on loam soil. Statistical comparison of treatments was performed for each day (Table 2).

There were significantly higher NH_3_ concentrations in the headspace from volatilization of Urea-200 than equivalent rate of both PCU and control treatments on 23 of the first 26 days (d 1, 5–26; Fig 3 and Table 2). Ammonia concentrations from Urea-200 were also significantly higher than PCU-200 on day 2, but similar to the control. All NH_3_-N concentrations returned to background levels beginning on day 31 and there were no significant differences among N sources thereafter. In this 45-Day Study, NH_3_ volatilization from PCU was statistically higher than from uncoated urea on days 4 and 27–30 (Fig 3, Table 2).

**Fig 4:**
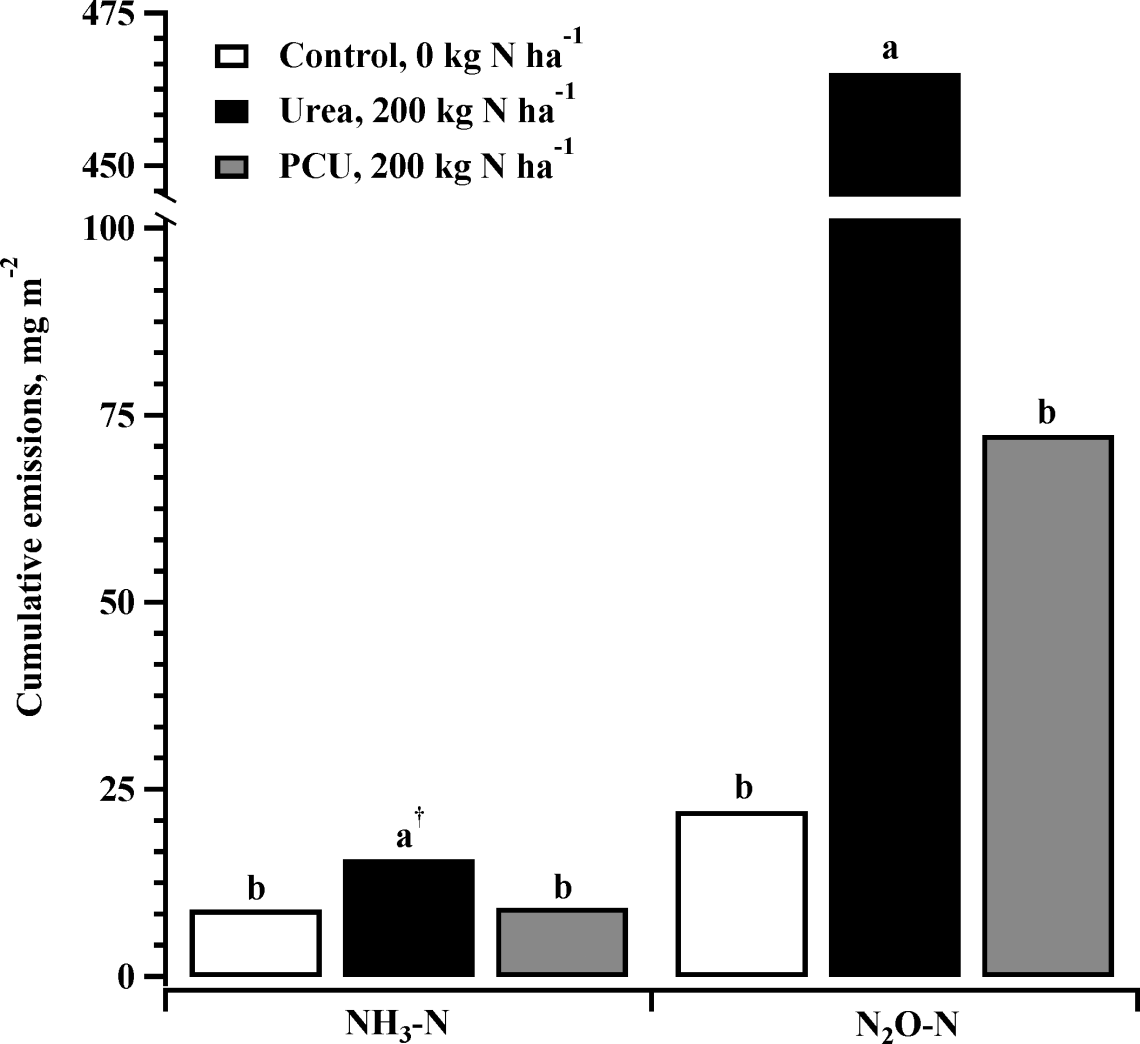
Estimated total emissions of NH_3_ and N_2_O from soil fertilized with either uncoated urea or polymer coated urea (PCU) and compared to an untreated control in a 45 d study with loam soil.

Cumulative N loss from NH_3_ volatilization was also significantly higher for Urea-200 than both the PCU-200 and control treatments at the *P* = 0.0713 level (Fig 4) and was equal for PCU and the control treatments (*P* = 0.9320). Fertilization with 200 kg N ha^-1^ PCU resulted in reduction of N losses from NH_3_ volatilization of 42% compared to 200 kg N ha^-1^ urea (Fig 4). The reduction rates for both 20-day and 45-day studies are similar to those found by others [37,38]. This reduction may be attributed to the controlled N release by the polymer coating, so that the N supply matches the biological demand.

### Nitrous oxide

#### 20-Day studies

As with NH_3_, the fertilizer source by time interaction was significant and, therefore, daily measurements of N_2_O concentrations are presented for each treatment for the three soils (Fig 1).

Sand Study–Unlike NH_3_ volatilization and N_2_O in all other studies, there was no day by source interaction for N_2_O emissions in the Sand study. As with NH_3_, the nearly biologically inert sand produced N_2_O evolution near background levels for all treatments (Fig 1), and no significant differences were observed over the course of the study (Table 2).

Sandy Loam Study–There were significantly higher N_2_O concentrations in the headspace above the soil treated with both rates of urea compared to the PCU and control treatments on all days except d 2 in the sandy loam soil (Table 2, Fig 1). Concentrations of N_2_O were significantly higher for Urea-336 than Urea-224 only once on d 5 and only three times for PCU-224 over the control on d 1, 5, and 6. Nitrous oxide evolution was never greater for PCU-224 than either urea treatment.

Cumulative N_2_O-N losses from Urea-336 were equal to the Urea-224 in the Sandy Loam study (*P* = 0.7768, Fig 2). Cumulative N_2_O-N losses from these two rates of urea were significantly greater than the control, and nearly significantly greater than PCU-224 (P = 0.0955 and 0.0606, respectively). Cumulative N_2_O losses for control and PCU treatments were equivalent (*P* = 0.5563). Fertilization with 224 kg N ha^-1^ PCU instead of 224 kg N ha^-1^ urea resulted in a 40% reduction of N_2_O emissions (Fig 2).

Loam Study–As with data for NH_3_ volatilization, data for days 1–4 of the Loam study are omitted from statistical analyses due to known analytical errors encountered due to contamination occurring during repair of some chambers. However, there were significantly higher N_2_O concentrations in the headspace above both urea treatments than the PCU-224 and control all other days of the study (Fig 1, Table 2). The N_2_O concentrations from Urea-336 were also significantly higher than Urea-224 on all days of the study except d 5. The PCU and control were statistically equivalent until d 18, after which N_2_O evolution from PCU-224 was significantly higher than the control until the end of the study (Fig 1, Table 2).

Cumulative N_2_O-N losses from the two urea treatments in the Loam study were statistically equivalent (Fig 2; *P* = 0.3359). Urea-336 evolved significantly more N_2_O-N than either the PCU or control treatments (*P* = 0.0137 and 0.0138, respectively). PCU-224 was equivalent to Urea-224 and the control. Fertilization with 224 kg N ha^-1^ PCU rather than 224 kg N ha^-1^ urea resulted in a 61% reduction of N_2_O emissions in the loam study (Fig 2).

#### 45-Day study

There were significantly higher N_2_O concentrations in the headspace from Urea-200 compared to the control on all but d 1 of the study (Fig 3, Table 2). On d 1–2, PCU-200 emitted less N_2_O than both the control and Urea-200 (Table 2), and d 2 emissions from PCU-200 were lower than both the Urea-200 and the control, although these differences were not practically significant (Fig 3). From d 3–11 (early period of the study), N_2_O emissions for PCU-200 were also significantly higher than the control, yet also significantly lower than Urea-200 application (Fig 3).

Cumulative N_2_O-N loss from urea was significantly larger than both PCU and control treatments (Fig 4; *P* < 0.0001) while PCU and control treatments were equivalent (*P* = 0.6261). Application of equivalent levels of PCU and urea (200 kg N ha^-1^) resulted in an 87% reduction of N_2_O emissions from PCU compared to urea. Similar reductions have been measured in corn cropping systems [38]. Similar to the NH_3_ volatilization reductions, likely result from improved coordination of N supply and N utilization by plants.

In the 20-Day Loam study, emissions of N_2_O from PCU increased slightly in the last three days of the trial. Other researchers have reported high N_2_O emissions from PCU but later than from urea [21]. Our 45-Day Study was conducted to ascertain whether increased N_2_O flux would occur after 20 d by using a similar PCU as with the 20-Day Studies except with a shorter-release time PCU (Duration CR45; 45-d release time). There were slightly but significantly elevated N_2_O emissions from PCU compared to the control from d 3 through 11 but not during the 12- to 45-d period. Nitrous oxide flux from urea-treated pots increased beginning on d 4, reached a peak after 15–16 d, and decreased slightly each day thereafter, but never reaching background levels even after 45 days (Fig 3). From our four studies, it is clear that N_2_O evolution from applied PCU is minimal compared to urea—even over the full 45-d release period for which this PCU was designed (Figs 1 and 3).

Halverson et al. performed a multi-year study on irrigated no-till maize cropping system and clay loam soil in which they compared readily soluble dry granular urea to “enhanced efficiency” N fertilizers, including PCU (ESN). They used the static-chamber method for gas collection and a gas chromatograph (GC) to analyze the evolved gases. They reported similar significant decreases in N_2_O emissions from PCU compared to urea [21]. However, unlike their study, we observed differences between control and PCU in the first 3–20 days, and there was no noticeable peak in flux from PCU to suggest a flush of N into the soil solution. Rather, levels of N_2_O were only slightly higher than the control for most of the study and generally not significantly different from the control.

Our results also support the findings of those who have documented decreased emissions of N_2_O from fertilization with PCU compared to uncoated urea in barley (*Hordeum vulgare* L.), cabbage (Brassica oleracea L.), maize, potato (*Solanum tuberosum* L.), and ryegrass (*Lolium perenne* L.) [16,21,29,39–41]. Ours is the first to observe these N_2_O fluxes using PAIRS analysis and to provide more than a snapshot of N_2_O flux following fertilization. In other studies, N_2_O flux was measured at most daily and more typically 2–3 times week^-1^ and flux estimated via linear interpolation. Mosier et al. (1991) suggested that sampling frequency be increased to account for the innately large temporal variability associated with the biologically mediated flux of N_2_O [31]. Venterea et al. (2009) observed a large variability in the accepted static chamber/GC method and suggested ways to minimize the inevitable variance [30]. This study demonstrates that by using PAIRS analysis, temporally high-resolution measures can be made and utilized to observe the gaseous losses of N following fertilization.

There were no noticeable differences in plant health throughout the duration of these studies between PCU and urea. This further supports the findings of others that use of PCU is at least equal to urea in terms of plant health. In fact, in many instances, crop yield and/or quality are improved [1,3,13–16,19–22,42].

## Conclusion

While providing an adequate N supply and maintaining plant health, PCU treatments were able to decrease N_2_O and NH_3_ evolution significantly compared to urea. The large decreases in the emissions of the potent greenhouse gas N_2_O and the atmospheric reactive NH_3_, while adequately supplying N to the plant, are major improvements to NUE. Our data suggest that PCU is a viable option to minimize loss of N following fertilizer application. Nitrogen losses from urea as NH_3_ and N_2_O were reduced by an average of 45 and 40%, respectively. Utilizing PAIRS analysis with non-static, non-flow through chambers provided a temporally high-resolution continuous and simultaneous viewpoint of both N_2_O and NH_3_ emissions. This research indicates that this method is useful, effective, and likely more accurate than methods employing far fewer sampling times, but additional research is needed to assess the variability associated with this method and to design a chamber to allow for relatively easy continuous field sampling.

In many areas of intensive agricultural production, fertilization is the leading contributor to anthropogenic pollution of reactive nitrogen (ammonia) being volatilized into the atmosphere with subsequent deposition into potentially sensitive terrestrial and aquatic ecosystems. And, the very potent greenhouse gas nitrous oxide emissions are significant from fertilization. The impacts of these pollutants can be catastrophic and need immediate action. The significance of the findings of this research have global ramifications in at least two ways. First, our data shows that PCU has the potential to greatly reduce environmental losses of N to the atmosphere. Secondly, the methodology developed represent an improved methodology which allows for accurate assessment of fertilizers. Traditional methods of doing this are performed in closed system, which are not representative of actual soil-plant-atmosphere conditions, and typically only are measured one or two times per day or week. Our system measures nearly continuously in an open system—providing a potential improvement in accuracy.

## Supporting information

S1 Dataset Herein can be found the raw data used to generate the figures and statistics for this publication. (XLSX)
